# Retrospective Three-Dimensional CT Reconstruction in Complex Emergency Hepato-Pancreato-Biliary Surgery: A Proof-of-Concept Case Series with Anatomical Concordance to Intraoperative Findings and Narrative Review

**DOI:** 10.3390/jcm15156028

**Published:** 2026-08-03

**Authors:** Horea-Florin Bocșe, Dana Monica Iancu, Paul-Andrei Ștefan, Codruța Gherman-Lencu, Alin Cornel Fetti, Nadim Al Hajjar, Florin Vasile Zaharie

**Affiliations:** 1 Department of Surgery, Faculty of Medicine, “Iuliu Hațieganu” University of Medicine and Pharmacy, Victor Babeș Street, No. 8, 400012 Cluj-Napoca, Romaniaconfetti_ro@yahoo.com (A.C.F.); na_hajjar@yahoo.com (N.A.H.); florinzaharie@yahoo.com (F.V.Z.); 2 “Octavian Fodor” Regional Institute of Gastroenterology and Hepatology, Croitorilor Street, No. 19-21, 400162 Cluj-Napoca, Romania; 3 Department No. 1, Fundamental Disciplines, Faculty of Nursing and Healthcare Sciences, “Iuliu Hațieganu” University of Medicine and Pharmacy, Victor Babeș Street, No. 8, 400012 Cluj-Napoca, Romania; stefan_paul@ymail.com (P.-A.Ș.); codruta_lmc@yahoo.com (C.G.-L.); 4 Radiology and Imaging Department, County Emergency Hospital, Clinicilor Street, No. 3-5, 400006 Cluj-Napoca, Romania

**Keywords:** three-dimensional imaging, computed tomography (CT), emergency surgery, hepato-pancreato-biliary surgery, pancreaticoduodenectomy, aortoenteric fistula, vascular mapping, AI-assisted 3D reconstruction

## Abstract

**Background/Objectives**: Three-dimensional (3D) reconstruction of contrast-enhanced computed tomography (CT) data has shown value in elective hepato-pancreato-biliary (HPB) surgery, but its role in complex abdominal emergencies remains poorly defined. Complex emergency HPB operations are high-acuity procedures in which precise preoperative anatomical knowledge directly influences outcome, because inadequate characterization of vascular relationships in these operations may lead to intraoperative catastrophe, unrecognized vascular injury, incomplete resection, or perioperative death. We report a retrospective proof-of-concept case series designed to test whether 3D reconstructions generated from preoperative CT datasets, when read by an operator blinded to the intraoperative findings, correspond to the intraoperative findings used as ground truth and provide anatomical information beyond the original conventional CT report. **Methods**: Single-center retrospective consecutive case series of five patients who underwent complex emergency HPB surgery at the Regional Institute of Gastroenterology and Hepatology “Prof. Dr. O. Fodor,” Cluj-Napoca, Romania, between July 2018 and July 2022. All operations were guided by conventional contrast-enhanced CT. Three-dimensional reconstructions were generated after surgery, from the archived preoperative CT datasets by a single reader who was blinded to the intraoperative findings, to the operative note, and to the histopathological diagnosis. The 3D reconstructions were compared qualitatively with the intraoperative findings (ground truth) and with the original radiological report. **Results**: Five patients (3 women, 2 men; mean age 62.2 years) were analyzed: a right colonic flexure tumor with duodenal invasion, a duodenal gastrointestinal stromal tumor (GIST), an aortoduodenal fistula, a complex bilioduodenal fistulization with right hepatic artery pseudoaneurysm, and a periampullary tumor in contact with the inferior vena cava (IVC). In all five cases the blinded 3D reconstruction corresponded closely with the intraoperative findings, whereas the original conventional CT report did not match the intraoperative ground truth as precisely—particularly with respect to the exact location, the longitudinal and circumferential extent, and the local topography of vascular involvement and of fistulous tracts. **Conclusions**: Under blinded retrospective comparison, post hoc 3D reconstructions—generated through independent reassessment of the archived CT imaging by a reader blinded to the intraoperative findings—corresponded more closely with intraoperative findings than the original same-day emergency CT report in depicting vascular topography across five complex emergency HPB operations. These hypothesis-generating findings motivate a prospective study of preoperative 3D reconstruction within the emergency imaging pathway.

## 1. Introduction

The use of three-dimensional (3D) image reconstruction in abdominal surgery has expanded in recent years, particularly in elective hepato-pancreato-biliary (HPB) and oncologic procedures. In contrast, its role in complex abdominal emergencies remains sparsely reported, with the literature largely limited to isolated case reports and small series. In the acute setting—where time pressure, distorted anatomy, and hemodynamic instability often coincide—the potential usefulness of rapid and accurate 3D visualization is compelling, but its exact contribution is yet to be clearly defined.

Complex abdominal emergencies—such as perforated tumors, severe intra-abdominal sepsis, fulminant pancreatitis, or extensive vascular involvement—are widely regarded as high-risk scenarios, in which both the therapeutic strategy and the operative technique are particularly challenging. In contrast to elective surgery, there is usually little time for thorough preoperative optimization, detailed multidisciplinary (MDT) discussions, or additional staged imaging. In this context, standard two-dimensional contrast-enhanced computed tomography (CT) may be difficult to interpret, and insufficient spatial orientation can further increase the risk of surgery. Three-dimensional reconstruction of imaging data has the potential to mitigate some of these issues by providing a more intuitive depiction of key anatomical structures, the true extent of disease, and the spatial relationships between pathological processes and adjacent organs or major vessels.

A meta-analysis by Somers and Bipat showed that contrast-enhanced CT has a summary positive predictive value of 81% for pancreatic resectability, indicating that a substantial proportion of patients may still undergo unnecessary surgical exploration [1]. Earlier periampullary studies explored the role of 3D-CT and CT angiography with 3D reconstruction, showing improved preoperative assessment of local tumor extent and vascular invasion [2,3]. In elective liver surgery, 3D reconstruction has been used predominantly in major or technically demanding hepatectomies, with improved intraoperative navigation and resection accuracy reported in approximately half of published studies [4]. In a multicenter prospective trial in hepatocellular carcinoma, the operative plan changed in 35% of cases after 3D review, while the discrepancy between the 3D-based plan and the procedure ultimately performed was only 18%, suggesting that 3D models may more closely predict the actual intraoperative situation than conventional two-dimensional imaging [5]. An international multicentric survey demonstrated that 3D reconstruction improved surgeons’ understanding of tumor–vessel relationships compared with conventional CT [6]. Comparative liver studies and meta-analyses have also reported shorter operative time, reduced blood loss, fewer complications, and improved recurrence-free outcomes, although the available evidence remains limited by heterogeneity and selection bias [7,8]. In pancreatic surgery, several studies have shown that 3D reconstructions improve visualization of vascular anatomy, may support safer pancreaticoduodenectomy (PD) by identifying arterial and biliary variants, and can improve the prediction of vascular invasion compared with conventional CT and magnetic resonance imaging (MRI) [9,10,11,12,13,14]. Current reviews nevertheless conclude that the pancreatic evidence is not yet sufficiently robust to support routine use of 3D reconstruction in all patients, and that stronger comparative studies are still required [15].

Two practical considerations frame the present work. First, complex emergency HPB operations are by nature rare: scenarios in which a time-critical emergency, a complex HPB pathology, and a preoperative contrast-enhanced CT of sufficient quality for 3D reconstruction coexist are encountered only sporadically, even in high-volume centers. Second, these operations are high-acuity procedures that require highly experienced HPB surgical teams; even expert surgeons rely on accurate preoperative anatomical maps, because inadequate characterization of vascular or fistulous relationships in the emergency setting can translate directly into unrecognized intraoperative injury, uncontrolled hemorrhage, incomplete resection, or perioperative death. Precise anatomical information is, in this population, a safety requirement rather than a refinement. Against this background, the published literature on 3D reconstruction in HPB surgery is dominated by elective cohorts [1,2,3,4,5,6,7,8,9,10,11,12,13,14,15,16], with only a handful of reports addressing the emergency setting specifically [17,18,19]. The present series contributes to this underreported domain.

Given the paucity of data on 3D reconstruction in emergency HPB surgery, and the fact that in our practice 3D reconstructions were not available in real time during the index operations, we designed the present study as a retrospective proof-of-concept/anatomical-validation case series. Our aim was to test whether 3D reconstructions generated retrospectively from the preoperative CT datasets, by an operator blinded to the intraoperative findings, corresponded to the intraoperative findings (used as ground truth), and whether they depicted critical anatomical relationships more clearly than the original conventional CT report. Our hypothesis was that the retrospective 3D reconstructions would accurately reproduce the intraoperative anatomy and would provide additional spatial information over conventional CT, particularly regarding the location, extent, and topography of vascular involvement, thereby justifying a prospective study of preoperative 3D reconstruction in complex emergency HPB surgery.

## 2. Materials and Methods

### 2.1. Study Design

This was a single-center retrospective consecutive case series, designed as a proof-of-concept/anatomical-validation study. The reporting follows the principles of the CARE guideline for case series.

### 2.2. Setting and Ethics

Patients were treated in the Department of Surgery of the Regional Institute of Gastroenterology and Hepatology “Prof. Dr. O. Fodor,” Cluj-Napoca, Romania, between July 2018 and July 2022. The study was approved by the Ethics Committee of the Regional Institute of Gastroenterology and Hepatology “Prof. Dr. O. Fodor”, Cluj-Napoca, and as well by the Ethics Committee of the University of Medicine and Pharmacy “Iuliu Hațieganu”, Cluj-Napoca.

### 2.3. Participants and Inclusion Criteria

During the study period (July 2018–July 2022), 15 patients underwent complex emergency HPB surgery at the Regional Institute of Gastroenterology and Hepatology “Prof. Dr. O. Fodor,” Cluj-Napoca, across all surgical teams. Of these, five operations were performed by the senior surgeon (D.I.) and her surgical team; the remaining ten were performed by other surgical teams within the department and fall outside the scope of the present single-surgeon series. The five cases reported here represent the complete, consecutive series of complex emergency HPB operations performed by D.I. during the study period. All five had an archived preoperative contrast-enhanced CT dataset of quality sufficient for retrospective 3D reconstruction. The cases are described as illustrative in that they happen to span a wide range of tumor–vessel, fistulous, and multivisceral anatomical complexity; this descriptive observation did not influence their inclusion, since all five of D.I.’s consecutive cases are reported. These five cases were not selected to constitute a disease-specific cohort; rather, they were chosen to test a single common question across a deliberately heterogeneous range of anatomical scenarios—whether retrospective 3D reconstruction reproduces the operative topography of a lesion’s relationship to adjacent major vessels and biliary structures—encountered in emergency HPB practice.

### 2.4. Conventional CT Protocol

All patients underwent multidetector contrast-enhanced CT in the emergency department on the scanner then in clinical use—Somatom Perspective (Siemens, Munich, Germany), with reconstructions available at 1.5–3 mm slice thickness and standard arterial and portal venous contrast phases; the same CT dataset was used for both the original radiological report and the subsequent 3D reconstruction. The original radiological interpretation was performed by the on-call radiologist and is referred to below as “the conventional CT report”.

### 2.5. 3D Reconstruction Workflow

All three-dimensional reconstructions were generated retrospectively, after the index operation, from the archived preoperative contrast-enhanced CT datasets using an AI-assisted 3D reconstruction platform. This platform is a surgical planning technology developed to convert conventional two-dimensional CT imaging into interactive, anatomically accurate, and surgically meaningful three-dimensional models, combining artificial intelligence–based segmentation with expert radiological validation to identify, isolate, and spatially render the anatomical structures most relevant to complex hepato-pancreato-biliary surgery.

The reconstruction workflow included importation of the arterial and portal venous CT phases, selection of the most informative phase for each anatomical target, and AI-assisted segmentation of organs, vessels, pathological lesions, and relevant fistulous or inflammatory structures. In each case, particular attention was given to structures with direct operative relevance, including the hepatic artery, portal venous system, superior mesenteric vessels, inferior vena cava, aorta, bile ducts when visible, duodenum, pancreas, liver, tumor boundaries, pseudoaneurysms, and fistulous tracts. The automatically generated segmentations were then reviewed and corrected when necessary, by M.S., a collaborating radiologist with expertise in HPB imaging and three-dimensional reconstruction, in order to ensure anatomical consistency, radiological accuracy, and surgical relevance. Because generating a 3D reconstruction requires direct examination of the archived axial and multiplanar source images (arterial and portal venous phases) in order to perform segmentation, this process constituted an independent reassessment of the original CT dataset itself, rather than a reformatting of the original radiology report; the reader did not have access to, or make use of, the text of the original report at any stage of this process.

The AI-assisted segmentation workflow required only minimal manual correction in the majority of cases; occasional manual refinements, mainly to optimize delineation of specific anatomical structures or vascular contours, generally required no more than approximately 10 min per case. The average reconstruction time was approximately 7 min, though this is dependent on CT dataset quality and on the computational performance of the reconstruction workstation.

The aim of this workflow was not limited to producing a visual three-dimensional rendering of the CT dataset. Rather, the objective was to generate a preoperative surgical map emphasizing the topography of disease in relation to major vascular, biliary, and visceral structures. The 3D models were therefore analyzed with specific attention to the location of the lesion, the longitudinal and circumferential extent of vascular contact, the distinction between vascular compression and suspected invasion, the displacement of adjacent vessels, the course and extent of fistulous tracts, and the relationship between pathological structures and potential dissection planes.

In the present study, the reader who generated and interpreted the 3D reconstructions was blinded to the intraoperative findings, to the operative note, and to the histopathological diagnosis. The reader had access only to the archived preoperative contrast-enhanced CT dataset and to the basic clinical indication for surgery. This design was chosen to reduce confirmation bias and to assess whether AI-assisted 3D reconstruction could reproduce the anatomical findings encountered intraoperatively without prior knowledge of the surgical outcome.

Although the reconstructions were performed retrospectively in this proof-of-concept series and were not available to the surgical team during the index operations, this workflow was conceived as a model for future prospective emergency implementation. In such a setting, the clinical value of AI-assisted reconstruction would depend not only on anatomical accuracy, but also on turnaround time, integration with the hospital imaging pathway, and the ability to provide surgeons with a clear and actionable anatomical representation without delaying urgent operative management. This is particularly relevant in complex emergency HPB surgery, where rapid understanding of vascular involvement, fistulous anatomy, and multivisceral relationships may directly influence operative strategy and patient safety.

### 2.6. Comparison with Intraoperative Findings

For each case, the 3D reconstruction was compared qualitatively with (i) the intraoperative findings recorded in the operative note and, where available, in intraoperative photographs, used as the anatomical ground truth, and (ii) the original conventional CT report. Concordance was assessed in terms of the identification, topography, and extent of the critical lesion and its relationship to adjacent vessels, bile ducts, and viscera. Concordance was assessed retrospectively using five predefined anatomical domains, applied as relevant to each case: (1) anatomical location, (2) longitudinal extent, (3) circumferential extent, (4) distinction between vascular compression and invasion, and (5) course and extent of fistulous tracts. Each domain was scored as concordant, partially concordant, or discordant with the intraoperative ground truth. The intraoperative ground truth was extracted from the operative notes and intraoperative photographs by a co-author (H.B.), who was blinded to the 3D reconstruction findings at the time of extraction; the 3D reconstructions were generated and interpreted by a separate reader (M.S.) blinded to the intraoperative findings. The comparison is therefore bidirectionally blinded. Because the 3D-reconstruction reader was blinded to the intraoperative findings, confirmation bias was formally mitigated in the interpretation of the 3D models. Inter-rater agreement was not assessed, as a single reader was used; this is acknowledged as a limitation. The 3D reconstruction reader independently reassessed the full archived CT dataset, blinded to the intraoperative findings; the comparator against which this reassessment was evaluated for the purposes of this study, however, was the original conventional CT report produced by the on-call radiologist during emergency care, rather than a second, independently produced 2D radiological report using predefined criteria.

### 2.7. Variables and Follow-Up

The following variables were retrieved from the electronic medical records and anonymized with a numerical identifier: demographic characteristics (age, sex, area of residence); indication for emergency admission; preoperative laboratory and imaging investigations; final diagnosis; surgical procedure; histopathological diagnosis of the resected specimen; postoperative complications (graded according to the Clavien–Dindo classification); and long-term follow-up. Follow-up evaluations were performed at 3, 6, and 12 months postoperatively and yearly thereafter.

A summary of patient, procedural, and imaging characteristics is provided in Table 1.

## 3. Results

Between July 2018 and July 2022, five patients (3 women, 2 men; mean age 62.2 years; range 59–67) underwent complex emergency HPB surgery and were included in the analysis. Case-by-case descriptions are given below; a concise synthesis is presented in Table 1.

Across the five cases, the blinded 3D reconstruction was concordant with the intraoperative ground truth in every anatomical domain assessed as applicable to that case (location, longitudinal extent, circumferential extent, the compression-versus-invasion distinction, and/or fistula course, as relevant). In every case, the original conventional CT report, although it had identified the broad diagnosis, did not specify one or more of these domains with comparable precision (Table 2). The case-by-case narratives below detail this pattern.

### 3.1. Case 1—Right Colonic Flexure Tumor with Duodenal Invasion

**Figure 1:**
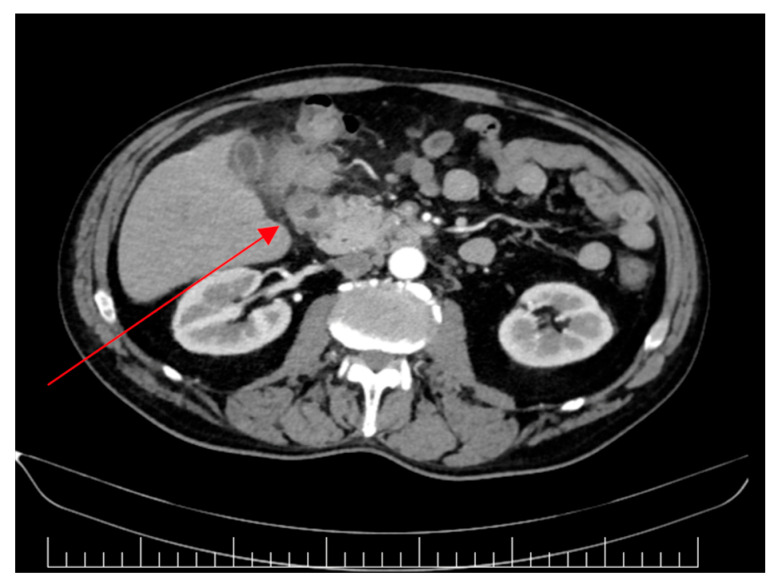
Conventional CT showing tumor invasion (infiltration) of the duodenum (red arrow).

**Figure 2:**
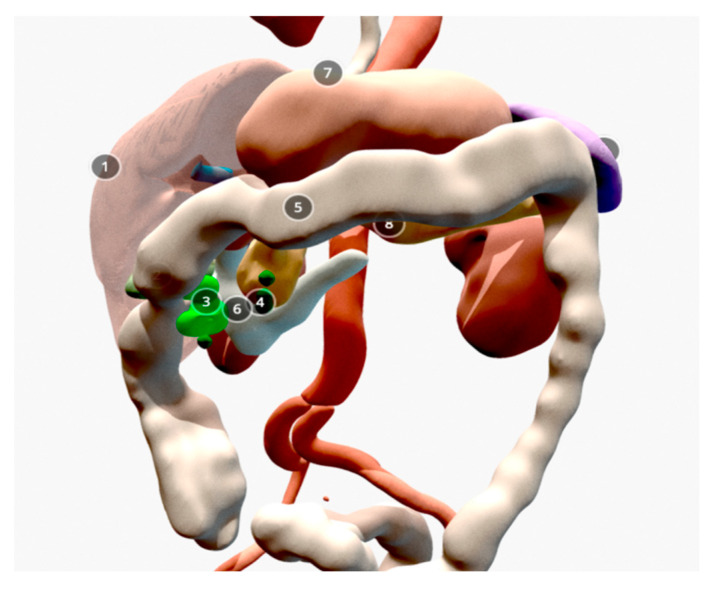
3D reconstruction showing right colon flexure tumor (3) with duodenal (6) invasion. Pancreas (4), Transverse colon (5), Liver (1), Stomach (7).

**Figure 3:**
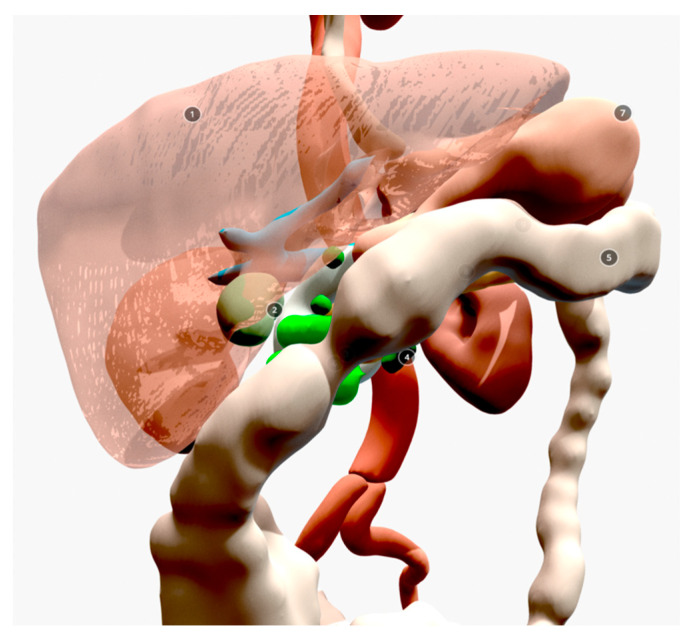
3D reconstruction showing the right colon tumor posterior invasion (green) of the duodenum. Transverse colon (5), Gallbladder (2), Pancreas (4).

A 67-year-old man was admitted with a stenotic tumor of the right colonic flexure and recurrent episodes of bowel obstruction. Contrast-enhanced CT demonstrated tumor invasion of the third and fourth portions of the duodenum (D3–D4) (Figure 1). The retrospective 3D reconstruction (Figure 2 and Figure 3), compared with the intraoperative findings, confirmed the extent of D3–D4 invasion and the relationship of the tumor to the superior mesenteric vessels. In blinded retrospective review, the 3D reconstruction corresponded with the intraoperative finding of circumferential D3–D4 invasion and with the preserved superior mesenteric vessels, whereas the original conventional CT report had not specified the longitudinal extent of duodenal invasion or its exact relationship to the superior mesenteric axis.

Pancreaticoduodenectomy with right hemicolectomy was performed. The early postoperative course was uneventful, with a 4-day intensive care unit (ICU) stay. The patient developed delayed gastric emptying (approximately 1000 mL/day of gastric stasis), which resolved within 7 days under conservative management (Clavien–Dindo grade II). He was discharged on postoperative day 10 without major complications. Follow-up during the first 2 postoperative years was uneventful.

### 3.2. Case 2—Duodenal Gastrointestinal Stromal Tumor (GIST)

**Figure 4:**
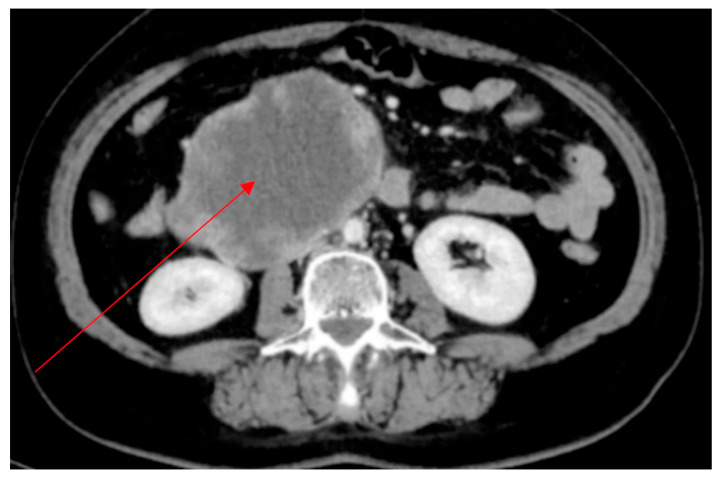
Conventional CT scan performed in the emergency setting, showing a large duodenal tumor (red arrow).

A 59-year-old woman presented with severe anemia (hemoglobin 6 g/dL) and recurrent episodes of upper gastrointestinal bleeding. Upper gastrointestinal endoscopy identified two actively bleeding ulcerated lesions in the second portion of the duodenum (D2), with surrounding mucosal infiltration suggestive of intramural tumor extension; endoscopic hemostasis was attempted, and biopsies were obtained. Contrast-enhanced CT revealed a large mass measuring 13 × 8 × 11.5 cm involving the D2–D3 segments, with extrinsic compression of the superior mesenteric vein (SMV) and the inferior vena cava (IVC), without definite radiological evidence of vascular invasion (Figure 4). A gastrointestinal stromal tumor (GIST) was suspected.

**Figure 5:**
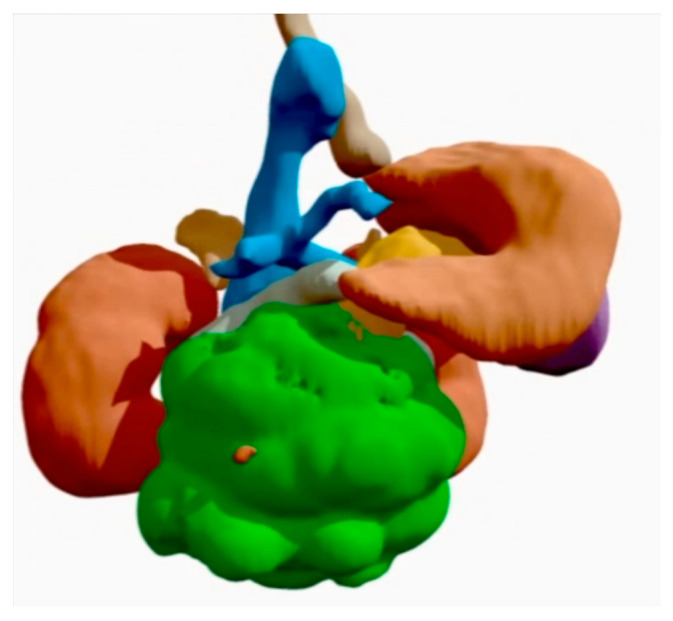
3D reconstruction showing the large tumor (green) and the extension to surrounding tissues.

The retrospective 3D reconstruction better delineated the interface between the tumor and the adjacent major vascular structures; it confirmed compression without invasion of the SMV and IVC, in agreement with the intraoperative findings (Figure 5). In blinded retrospective review, the 3D reconstruction was concordant with the intraoperative ground truth in the domains of location (D2–D3), circumferential extent of vascular contact, and the compression-versus-invasion distinction (extrinsic compression of the IVC and SMV without wall invasion), whereas the original conventional CT report had characterized the tumor–vessel interface only as “without definite vascular invasion,” without specifying circumferential extent or the displacement pattern of the superior mesenteric artery.

**Figure 6:**
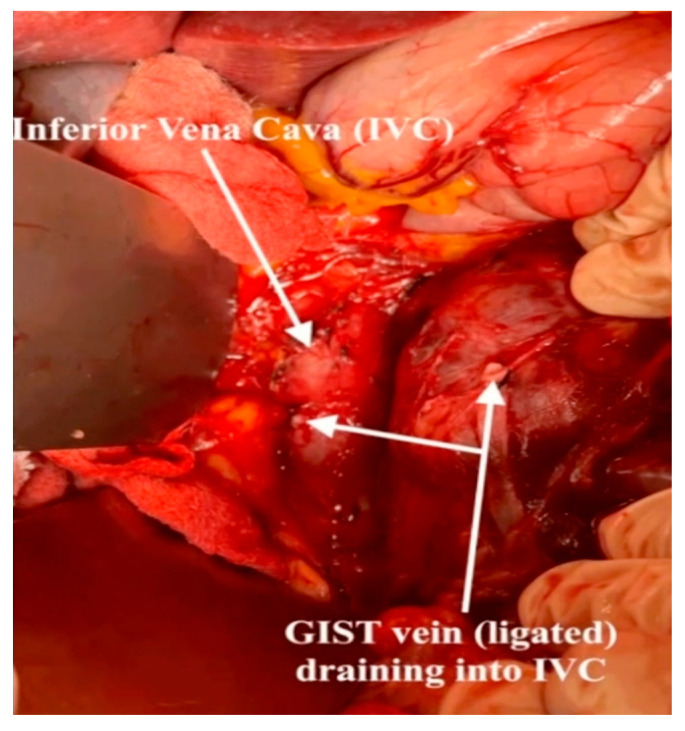
Intraoperative aspect of tumor resection. Close proximity to the IVC. Venous drainage of the tumor highlighted.

Pancreaticoduodenectomy with pancreatogastrostomy was performed without major intraoperative events; a 13-cm tumor in close proximity to the IVC was confirmed intraoperatively, without evidence of vascular invasion (Figure 6). During the first postoperative week, the patient developed a grade A postoperative pancreatic fistula after pancreaticogastrostomy which was managed conservatively; she was discharged after 2 weeks. Final histopathological examination confirmed a duodenal GIST (D2–D3 primary site; largest dimension 13 cm (13/10/5 cm); mitotic count [<5 mitoses/5 mm^2^]; high risk category according to the modified NIH/AFIP stratification; resection margins negative, R0). Follow-up during the first 2 postoperative years was uneventful, with adjuvant imatinib.

### 3.3. Case 3—Aorto-Duodenal Fistula

A 62-year-old man presented to the emergency department in severely altered general condition, with intense epigastric pain, pallor, and hemorrhagic shock, although he remained conscious. Laboratory investigations showed profound anemia (hemoglobin 3.5 g/dL). Immediate volume resuscitation was initiated, and once the patient’s condition allowed, contrast-enhanced CT was performed. Imaging demonstrated close adherence of the D3 segment to the descending aorta, associated with mural thrombus and active bleeding, raising strong suspicion of an aorto-duodenal fistula.

**Figure 7:**
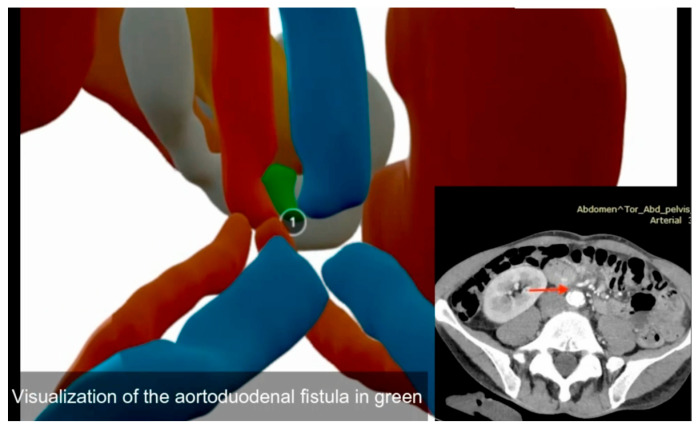
3D reconstruction showing the fistula (1—green) between the aorta (red) and duodenum (grey). Contrast enhanced CT (CECT) compared to 3D reconstruction (right) highlighting the fistula (red arrow).

The retrospective 3D reconstruction demonstrated the communication between the D3 segment of the duodenum and the descending aorta, the cranio-caudal extent of the aortic segment requiring resection relative to the renal arteries above and the iliac bifurcation below, and the distribution of the mural thrombus (Figure 7). In blinded retrospective review, the 3D reconstruction was concordant with the intraoperative ground truth in the domains of location (focal aorto-duodenal communication in the D3 segment), longitudinal extent (infrarenal aortic segment terminating above the iliac bifurcation), and fistula/thrombus course (mural thrombus distribution), none of which were specified with comparable precision in the original conventional CT report—features that directly inform the choice of open versus endovascular repair and the level of aortic cross-clamping.

**Figure 8:**
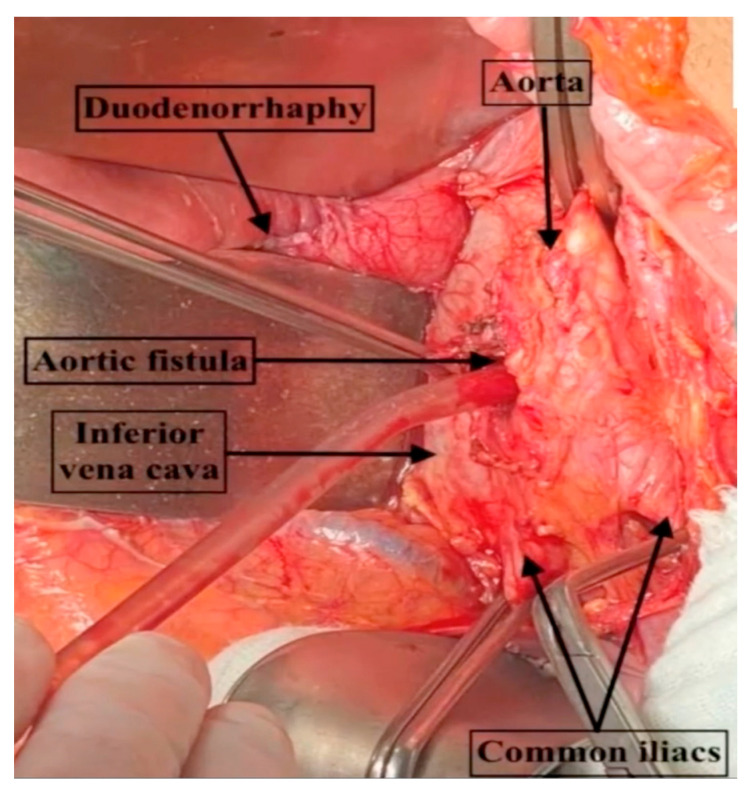
Intraoperative findings—Aortic fistula, after duodenal mobilization and duodenorrhaphy. Canula pointing the aortic fistula.

**Figure 9:**
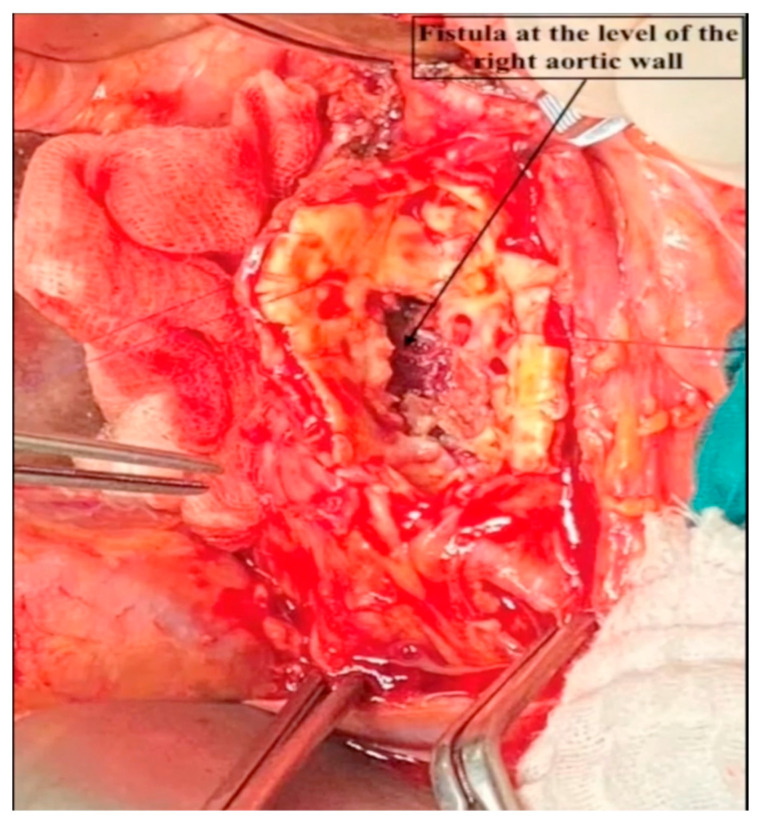
Intraoperative findings—Aortic fistula localization after clamping and sectioning. Aortic wall calcification.

The procedure was performed by a multidisciplinary team consisting of a general surgeon and a cardiovascular surgeon. After mobilization by a Kocher maneuver, partial resection of the D3 segment was carried out (Figure 8 and Figure 9). The aorta was cross-clamped below the renal arteries and above the iliac bifurcation, followed by resection of the involved descending aortic segment and reconstruction with a Dacron graft.

The initial postoperative course was favorable under anticoagulant and antiplatelet therapy. Two weeks after surgery, however, the patient developed irreversible ischemia of the left foot, which required amputation. The patient subsequently died 1 month postoperatively from complications following left foot amputation, related to the peripheral ischemia that developed after aortic cross-clamping and reconstruction; there was no evidence of failure or recurrence of the aortoduodenal fistula repair itself (Clavien–Dindo V).

### 3.4. Case 4—Complex Bilioduodenal Fistulization with Right Hepatic Artery Pseudoaneurysm

**Figure 10:**
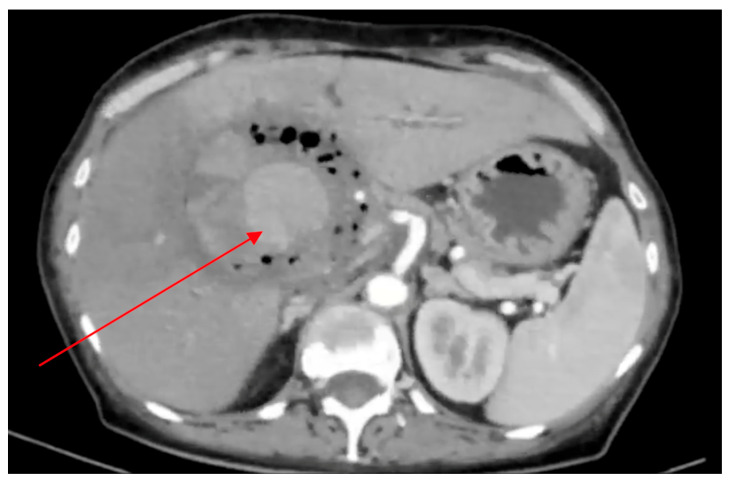
Conventional CT showing the intrahepatic abscess/gallbladder fistula—red arrow.

A 60-year-old woman presented to the emergency department with severe epigastric pain and vomiting. On admission, she was conscious and hemodynamically stable. Contrast-enhanced CT demonstrated a perforated bulbar ulcer associated with a fistulous communication involving the gallbladder and the common bile duct; in addition, a pseudoaneurysm of the right hepatic artery was identified (Figure 10).

**Figure 11:**
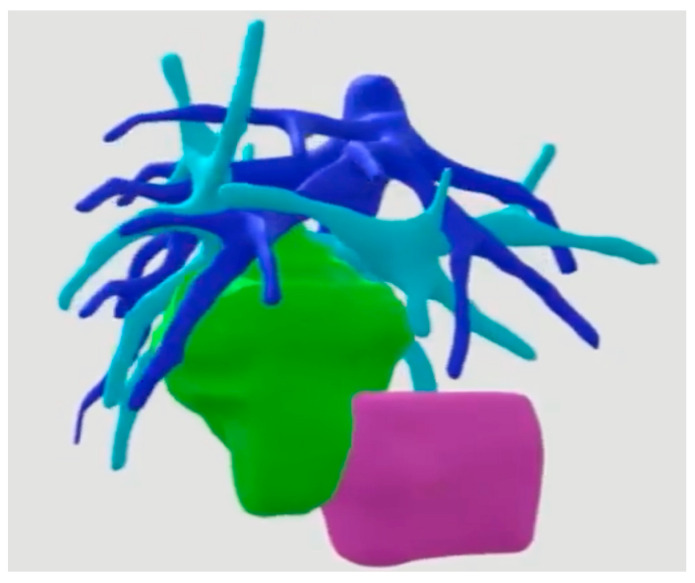
3D reconstruction showing the fistula/abscess relationship with the vessels (dark blue—hepatic veins, light blue—portal veins, purple duodenum, green abscess).

The retrospective 3D reconstruction (Figure 11) confirmed the presence and extent of the fistulous tract between the gallbladder and the common bile duct, showing involvement of the entire extrahepatic biliary tree, and further characterized the right hepatic artery pseudoaneurysm and its relationship to the fistula. In blinded retrospective review, the 3D reconstruction was concordant with the intraoperative ground truth in the domains of fistula course/longitudinal extent (cholecysto-choledochal fistula extending to the hilar plate) and topographical relationship to the right hepatic artery pseudoaneurysm, neither of which was specified with comparable precision in the original conventional CT report—information that is directly relevant to anticipating intraoperative vascular control and to planning the biliary reconstruction.

**Figure 12:**
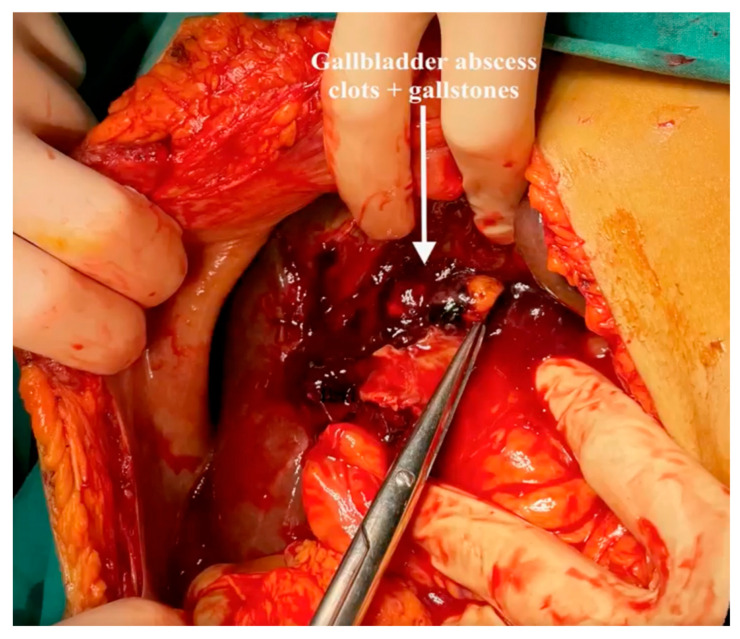
Intraoperative findings—gallbladder fistula with active bleeding and gallstones.

Surgical exploration revealed a perforated bulbar ulcer with a fistula between the gallbladder and the duodenum. During cholecystectomy, a large cholecysto-choledochal fistula involving the common bile duct up to the hilar plate was identified (Figure 12). Intraoperative dissection confirmed involvement of the right hepatic artery, with active bleeding encountered during fistula dissection; hemostasis was achieved with a 6-0 suture, and neither arterial resection nor vascular reconstruction was required. Because of the extensive biliary involvement, distal gastrectomy with Roux-en-Y portojejunostomy was performed. The postoperative course was favorable, without major complications; a low-output biliary fistula (approximately 300 mL/day, ISGLS grade A) developed postoperatively and was managed conservatively (Clavien–Dindo grade II). Final histopathological examination of the gastrectomy specimen unexpectedly revealed gastric adenocarcinoma, and the patient was referred to the MDT oncology board. At 1-year follow-up, the patient died from progression of the underlying gastric adenocarcinoma. No preoperative imaging or endoscopic finding, including the conventional CT, the 3D reconstruction, or upper gastrointestinal endoscopy, suggested a gastric malignancy; the 3D reconstruction in this case was directed at, and confined to, the fistulous and vascular anatomy (the cholecysto-choledochal fistula and the right hepatic artery pseudoaneurysm), and was neither designed nor expected to provide oncologic or histological characterization of the stomach.

### 3.5. Case 5—Periampullary Tumor with Inferior Vena Cava Contact

**Figure 13:**
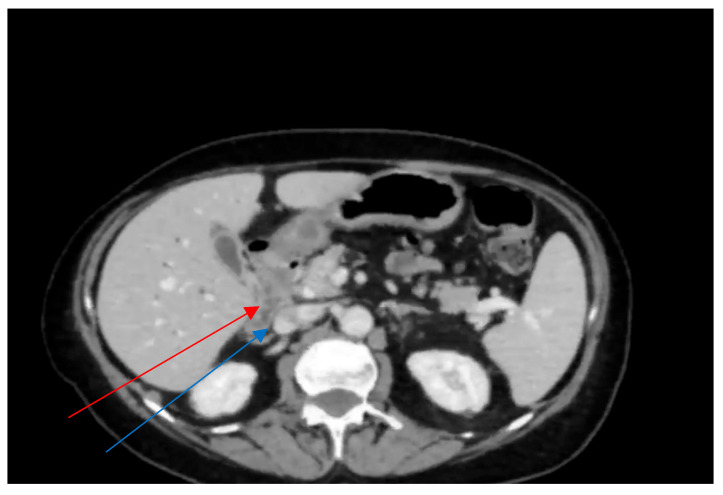
Conventional CT showing close contact of the tumor (red arrow) with the IVC (blue arrow).

A 63-year-old female patient presented with a bleeding periampullary tumor diagnosed on upper gastrointestinal endoscopy and on contrast-enhanced CT and was referred for pancreaticoduodenectomy. Endoscopy demonstrated an ulcerated ampullary lesion with active oozing, and biopsies were obtained. Standard preoperative CT showed no evidence of distant metastases and no other associated complications; close contact between the tumor and the inferior vena cava (IVC) was present on the original CT, but the exact extent of the interface was not specifically emphasized in the conventional report (Figure 13).

**Figure 14:**
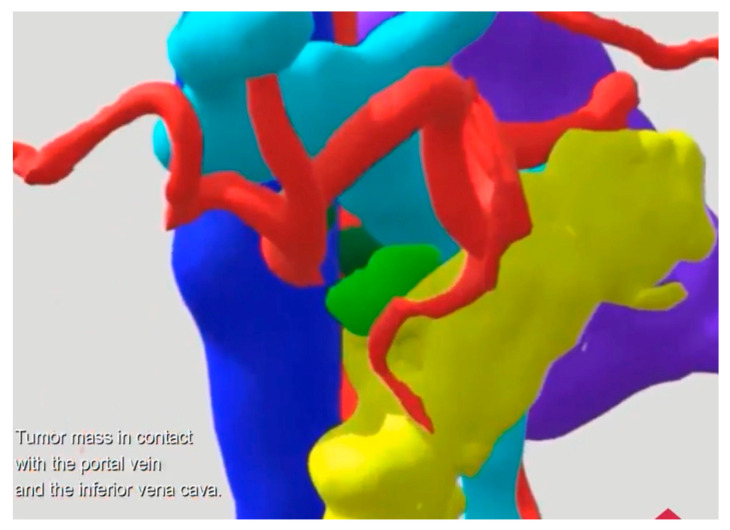
3D reconstruction showing close contact of the tumor (light green) with the IVC (posterior-dark blue).

In blinded retrospective review, the 3D reconstruction (Figure 14) was concordant with the intraoperative ground truth in the domains of location and longitudinal/circumferential extent of the tumor–IVC interface (requiring tangential caval resection); the original conventional CT report had mentioned the contact only in generic terms—information that the 3D model depicted explicitly.

**Figure 15:**
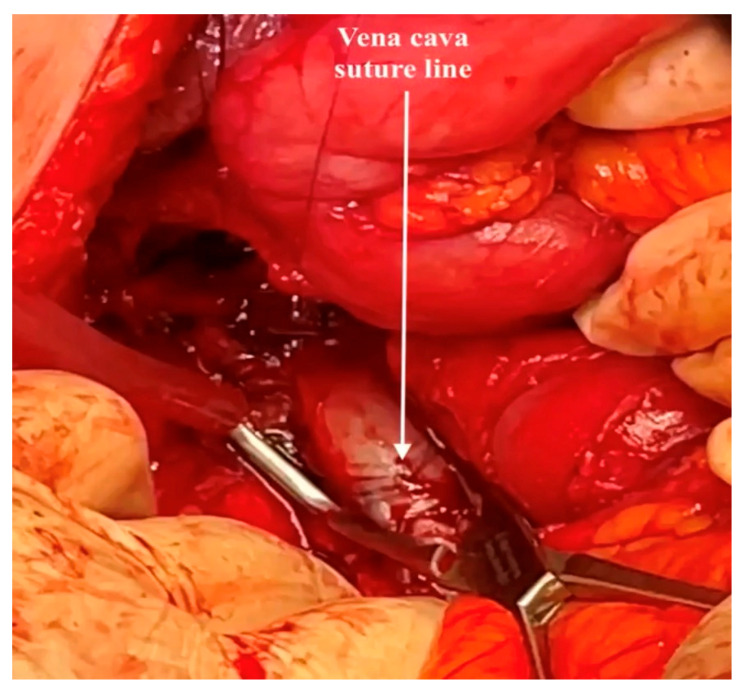
Intraoperative aspect of inferior vena cava suture line.

Intraoperatively, the close relationship between the tumor and the IVC was confirmed, and tangential caval resection with primary suturing was required (Figure 15). No major intraoperative or postoperative complications occurred (Clavien–Dindo grade I). Final histopathological examination revealed stage IB ductal adenocarcinoma (R0). Follow-up at 1 year was favourable.

## 4. Discussion

In this retrospective proof-of-concept series of five complex emergency HPB operations, post operative 3D reconstructions generated from the preoperative CT datasets by a reader blinded to the intraoperative findings corresponded closely with the intraoperative findings used as the anatomical ground truth. Under this blinded retrospective comparison, 3D reconstruction corresponded more closely with the intraoperative findings than the original conventional CT report in depicting the location, the longitudinal and circumferential extent, and the local topography of vascular involvement and of fistulous tracts in all five cases. Because the reconstructions were not available at the time of the surgery, no causal claim can be made that 3D guided surgical decision-making in these patients; the pattern observed is hypothesis-generating and supports a prospective evaluation in which the 3D model is available before the operative decision is taken.

These five cases are pathologically heterogeneous and are not presented as a disease-specific cohort. They were selected to test a single common question—whether retrospective 3D reconstruction reproduces the operative topography of a lesion’s relationship to adjacent major vessels and biliary structures—across the range of anatomical problems (neoplastic, fistulous, vascular) encountered in emergency HPB practice. No pathology-specific conclusion is intended or supported by this design.

The domain in which 3D reconstruction most consistently corresponded more closely with the intraoperative findings than the original conventional CT report was vascular topography—that is, the exact location of vascular involvement, the circumferential and longitudinal extent of that involvement, and the relationship of the index lesion to adjacent arterial and venous branches. This distinction is operationally important in emergency HPB surgery, because the choice between tangential and segmental caval resection, between infrarenal and suprarenal aortic cross-clamping, between primary repair and graft interposition, and between an open and a hybrid endovascular approach, is governed directly by these topographic features. A narrative radiological report describing “close contact,” “compression,” or “no definite invasion” compresses several distinct topographic scenarios into a single phrase; a volumetric 3D reconstruction preserves the distinction. In a time-critical setting, a model that renders these features immediately legible to the surgical team is likely to be more useful than the same information distributed across sequential axial slices.

Cross-walking our cases with the published literature, Case 1 and Case 5 illustrate the role of 3D reconstruction in periampullary and duodenal resection previously described by House et al. and Saldinger et al. [2,3], and more recently by Vicente et al. [13] and Rasenberg et al. [14], where 3D models may increase confidence in assessing local tumor extent and the relationship to the IVC and the superior mesenteric vessels. Case 2 echoes the pancreatic experience of Klauss et al., Horiguchi et al., and Miyamoto et al. [9,10,11], where 3D reconstruction clarifies the tumor–vessel interface and identifies arterial variants relevant to safe pancreaticoduodenectomy; the meta-analysis by Somers and Bipat [1] underscores why any incremental improvement in preoperative assessment is worth pursuing in this population. Case 3 (aortoduodenal fistula) and Case 4 (complex bilioduodenal fistulization with right hepatic artery pseudoaneurysm) extend the rationale articulated by Cotsoglou et al. [6] and Felli et al. [5] from elective hepatobiliary surgery into the emergency setting: when tumor–vessel relationships, active bleeding, or fistulization dominate the operative problem, a volumetric model may translate more directly into an operative plan than sequential axial slices, and the operative plan derived from 3D may more closely predict the actual intraoperative situation than conventional 2D imaging.

The published literature on 3D reconstruction in HPB surgery is dominated by elective cohorts [1,2,3,4,5,6,7,8,9,10,11,12,13,14,15,16], with only a small number of reports addressing the emergency setting specifically [17,18,19]. Aortoenteric fistula in particular has been described largely in case reports and small vascular-surgery series, with 3D reconstruction used sporadically and inconsistently [18,19]. High-complexity emergency HPB surgery outcomes are known to be sensitive to center volume, surgeon experience, and the quality of preoperative planning. The present series, although small, contributes directly to this underreported domain by demonstrating a consistent pattern under blinded retrospective comparison: namely, that 3D reconstructions generated post hoc from the preoperative CT datasets match the intraoperative ground truth more closely than the original conventional CT report, particularly with respect to vascular topography.

The stakes of inadequate preoperative anatomical information in these operations are not abstract. Complex emergency HPB operations are high-acuity procedures performed on patients who are often hemodynamically marginal, in whom the operative field is distorted by inflammation, hemorrhage, or fistulization. Even for experienced HPB teams, and even in high-volume centers, an incomplete understanding of the vascular topography before incision can translate into unrecognized vascular injury, uncontrolled bleeding, incomplete resection, or perioperative death. Precise preoperative mapping is, in this population, a safety requirement rather than a refinement. Our series does not prove that 3D reconstruction improves outcome; it suggests, under blinded retrospective comparison, that 3D reconstruction captures the anatomy the surgeon will encounter more faithfully than the original conventional CT report, and on that basis it argues that the modality warrants formal prospective evaluation in this high-acuity population.

Case 3 warrants specific comment, because the patient died at 1 month from complications of peripheral ischemia despite the 3D reconstruction having accurately depicted the aortoduodenal fistula and its vascular relationships. The informational benefit of 3D reconstruction is dissociable from the clinical outcome: the cause of death in this patient was a downstream complication of aortic reconstruction and antithrombotic therapy, not a failure of imaging strategy. This dissociation is important when interpreting any small series of emergency operations, in which outcomes are dominated by disease severity at presentation rather than by individual intraoperative adjuncts. Similarly, the 1-year death of the patient in Case 4 reflects the biology of an unexpectedly diagnosed gastric adenocarcinoma and is independent of the imaging adjunct evaluated in this study.

The practical feasibility of 3D reconstruction in the emergency pipeline deserves honest consideration. In an elective context, segmentation can be performed over hours or days, but in an emergency pathway the clinically relevant turnaround time is minutes to a few hours, and must not delay a hemodynamically unstable patient’s journey to the operating room. Key operational variables include out-of-hours availability of trained segmentation personnel, integration of the rendering software with the institutional picture archiving and communication system (PACS), the cost and licensing model of the software, and the requirement for standardized, robust acquisition protocols that survive the compromises of emergency imaging. Recent computer-aided and semi-automated workflows [12,14] suggest that turnaround times compatible with emergency care are achievable, but this has not yet been formally demonstrated at scale.

This study has substantial limitations. It is a retrospective single-center consecutive case series of only five patients with heterogeneous pathology, all operated by the same experienced surgical team. Because each of the five pathological categories in this series is represented by a single case, this study cannot determine whether 3D reconstruction offers differential anatomical benefit across pathology types (e.g., neoplastic vascular invasion vs. fistulous disease vs. hemorrhagic/aneurysmal pathology); this question requires a pathology-stratified prospective design with multiple cases per category. The small sample reflects the genuine rarity of emergency HPB scenarios in which a time-critical emergency, complex HPB pathology, and a preoperative contrast-enhanced CT of sufficient quality for 3D reconstruction coexist within a single institution; this rarity does not, however, diminish the statistical caveats that apply to any five-patient series. Three-Dimensional reconstructions were generated and interpreted by a single reader, blinded to the intraoperative findings, to the operative note, and to the histopathological diagnosis; confirmation bias was therefore formally mitigated. Inter-rater agreement was not assessed and the single-reader design is acknowledged as a limitation. Within this small series, we did not identify a clear instance of discordance between the 3D reconstruction and the intraoperative findings across the anatomical domains assessed. This should be interpreted cautiously: the assessment was retrospective, performed by a single reader without independent adjudication, and a series of five cases cannot meaningfully estimate a discordance rate. Formal assessment of concordance and discordance requires a larger, prospectively designed series with multiple independent readers. The 3D reconstruction reader independently reassessed the full archived CT dataset (axial and multiplanar arterial/portal venous phases) while blinded to the intraoperative findings, and this reassessment therefore constitutes a form of independent reinterpretation of the original imaging rather than of the original report alone. This reassessment was, however, performed by a single reader, was expressed only as a 3D reconstruction rather than as a second conventional-format 2D radiological report using a predefined checklist, and was not independently adjudicated by a second reader. We therefore cannot fully separate the anatomical benefit attributable to the 3D rendering itself from the benefit attributable simply to a second, unhurried, blinded review of the same source images by a different reader. No randomized or blinded second-radiologist comparator producing an independent 2D report was used, and we therefore make no claim regarding the effect of 3D reconstruction on operative time, blood loss, perioperative morbidity, or long-term survival. This is a single-surgeon consecutive case series: five of the 15 complex emergency HPB operations performed at the institution during the study period were performed by D.I., and all five are reported here in full, with no exclusions within her practice. This design eliminates within-series selection bias but limits generalizability, since surgeons or teams with different levels of experience, case volume, or institutional resources may encounter—and manage—complex emergency HPB pathology differently.

A prospective evaluation is warranted and could be designed as follows. Eligible patients would be adults admitted with a complex emergency HPB problem (active bleeding, suspected vascular involvement, complex fistulization, or anticipated multivisceral resection) in whom a contrast-enhanced CT is obtained. After the standard CT report, a pre-specified 3D reconstruction would be generated within a defined turnaround window (for example ≤ 2 h for hemodynamically stable patients, not applicable for unstable patients requiring immediate operative control). The primary endpoint would be a blinded change in operative plan attributable to 3D reconstruction, scored on a pre-specified typology (e.g., change in extent of resection, change in vascular strategy, change in reconstruction, change from open to hybrid approach). Secondary endpoints would include operative time, intraoperative blood loss, R0 rate, Clavien–Dindo complications, and 90-day mortality. Given the rarity of these scenarios within any single institution, a realistic sample-size estimate, assuming a change-of-plan rate of approximately 30% [5] and a minimal clinically meaningful absolute difference of 15%, would require approximately 150–200 patients accrued across 5–8 high-volume emergency HPB centers over a multi-year window.

Taken together, our findings suggest that CT-based 3D reconstruction may represent a useful adjunct in selected cases of complex emergency HPB surgery, particularly when major vascular structures, the biliary confluence, or multivisceral resection are involved. The present series does not demonstrate clinical utility; it demonstrates anatomical concordance under blinded comparison and, on that basis, motivates a properly designed prospective study.

## 5. Conclusions

In this retrospective proof-of-concept case series, post-surgery CT-based 3D reconstructions generated by a blinded reader corresponded more closely with the intraoperative findings than the original same-day emergency CT report in five complex emergency HPB operations, particularly in depicting the location, extent, and local topography of vascular involvement and of fistulous tracts; however, the single-reader, non-adjudicated design means this study cannot fully isolate the contribution of the 3D rendering itself from that of a second, unhurried blinded review of the same imaging. These findings do not establish clinical utility, but they support the hypothesis that preoperative 3D reconstruction could provide useful anatomical information in selected high-acuity emergency HPB scenarios—particularly when suspected vascular involvement, active bleeding, complex fistulization, or anticipated multivisceral resection are present. A prospective study with preoperative implementation, a pre-specified operative-plan endpoint, and a realistic assessment of turnaround time within the emergency pipeline is needed to determine whether this anatomical advantage translates into measurable improvements in perioperative and oncologic outcomes.

## Figures and Tables

**Table 1:** Patient, procedural, and imaging characteristics of the five cases.

Case #	Age/Sex	Presenting Complaint	Conventional CT Diagnosis	Additional Information from Retrospective 3D Reconstruction	Surgical Procedure	Post Operative Complications (Clavien–Dindo)	Follow-Up/ Outcome
1	67/M	Recurrent bowel obstruction from stenotic right colonic flexure tumor	Right colonic flexure tumor with D3–D4 duodenal invasion	Confirmed extent of D3–D4 invasion and relationship with superior mesenteric vessels. Under blinded comparison, 3D specified the longitudinal extent of duodenal invasion and the exact relationship to the superior mesenteric axis, which the conventional CT report had not characterized.	Pancreatico-duodenectomy + right hemicolectomy	Delayed gastric emptying (grade II)	Uneventful at 2 years
2	59/F	Severe anemia (Hb 6 g/dL); recurrent upper GI bleeding	Duodenal (D2–D3) mass 13 × 8 × 11.5 cm compressing SMV and IVC, without definite invasion	Clarified tumor–IVC/SMV interface; confirmed compression without invasion. Under blinded comparison, 3D explicitly depicted the circumferential extent of vascular contact, the absence of wall disruption, and the displacement of the SMA, resolving the non-specific “no definite invasion” wording of the conventional CT report.	Pancreatico- duodenectomy with pancreato- gastrostomy	Grade A postoperative pancreatic fistula (grade 0)	Uneventful at 2 years; duodenal GIST, R0, high risk (AFIP/NIH)
3	62/M	Hemorrhagic shock; epigastric pain; Hb 3.5 g/dL	Aorto-duodenal fistula (D3 adherent to descending aorta with mural thrombus and active bleeding)	Communication site, cranio-caudal extent of the aortic segment requiring resection, and mural thrombus distribution. Under blinded comparison, 3D specified the exact location of the aorto-duodenal communication, the infrarenal-to-supra-iliac extent of the involved aortic segment, and the longitudinal distribution of mural thrombus, none of which were specified in the conventional CT report.	Partial D3 resection; aortic resection with Dacron graft reconstruction (multidisciplinary)	Left foot ischemia → amputation at 2 weeks; death at 1 month (grade V)	Deceased at 1 month
4	60/F	Severe epigastric pain; vomiting	Perforated bulbar ulcer with cholecysto-choledochal fistula involving extrahepatic bile duct; right hepatic artery pseudo- aneurysm	Extent of fistulous tract; relationship of pseudoaneurysm to right hepatic artery. Under blinded comparison, 3D specified the longitudinal extent of the fistulous tract up to the hilar plate and the topographical relationship of the pseudoaneurysm to the right hepatic artery within the field of biliary dissection, neither of which was specified in the conventional CT report.	Cholecystectomy; distal gastrectomy with Roux-en-Y portojejunostomy	Low-output biliary fistula (ISGLS grade A; Clavien–Dindo grade II)	Gastric adenocarcinoma on histopathology; deceased at 1 year from disease progression
5	63/F	Bleeding periampullary tumor (endoscopy + CT)	Periampullary tumor in contact with IVC	Tumor–IVC interface depicted more conspicuously than in conventional CT report. Under blinded comparison, 3D specified the longitudinal and circumferential extent of the tumor–IVC interface, whereas the conventional CT report had described the contact only in generic terms.	Pancreatico- duodenectomy with tangential caval resection and primary suture	No major complications (grade I)	Ductal adenocarcinoma, stage IB, R0; favorable at 1 year

CT, computed tomography; GI, gastrointestinal; GIST, gastrointestinal stromal tumor; IVC, inferior vena cava; SMA, superior mesenteric artery; SMV, superior mesenteric vein; ISGLS, International Study Group of Liver Surgery.

**Table 2:** Case-by-case concordance between the blinded 3D reconstruction and the intraoperative ground truth across five predefined anatomical domains.

Case	Pathology	Location	Longitudinal Extent	Circumferential Extent	Compression vs. Invasion	Fistula/Thrombus Course
1	Colonic flexure tumor with duodenal invasion	Concordant	Concordant	N/A	N/A	N/A
2	Duodenal GIST	Concordant	N/A	Concordant	Concordant	N/A
3	Aorto-duodenal fistula	Concordant	Concordant	N/A	N/A	Concordant
4	Bilioduodenal fistulization with pseudoaneurysm	N/A	Concordant	N/A	N/A	Concordant
5	Periampullary tumor with IVC contact	Concordant	Concordant	Concordant	N/A	N/A

GIST, gastrointestinal stromal tumor; IVC, inferior vena cava; N/A, domain not applicable to that case’s pathology.

## Data Availability

The data presented in this study are available on reasonable request from the corresponding author. The data are not publicly available due to patient privacy restrictions.

## Abbreviations

The following abbreviations are used in this manuscript:

HPB	Hepato-Pancreato-Biliary
GIST	Gastrointestinal stromal tumor
AI	Artificial Intelligence
CT	Computed Tomography
3D	3 Dimensional
ISGLS	International Study Group of Liver Surgery
